# The integrative effects of behavior and morphology on amphibian movement

**DOI:** 10.1002/ece3.4837

**Published:** 2019-01-15

**Authors:** Evan M. Bredeweg, Anita T. Morzillo, Lindsey L. Thurman, Tiffany S. Garcia

**Affiliations:** ^1^ Department of Fisheries and Wildlife Oregon State University Corvallis Oregon; ^2^ Department of Natural Resources & the Environment University of Connecticut Storrs Connecticut; ^3^ Northern Rocky Mountain Science Center U.S. Geological Survey Bozeman Montana

**Keywords:** amphibian movement, behavioral variation, jump performance, life history, morphology, movement behavior

## Abstract

Animal movement and dispersal are key factors in population dynamics and support complex ecosystem processes like cross‐boundary subsidies. Juvenile dispersal is an important mechanism for many species and often involves navigation in unfamiliar habitats. For species that metamorphose, such as amphibians, this transition from aquatic to terrestrial environments involves the growth and use of new morphological traits (e.g., legs). These traits strongly impact the fundamental ability of an organism to move in novel landscapes, but innate behaviors can regulate choices that result in the realized movements expressed. By assessing the integrative role of morphology and behavior, we can improve our understanding of juvenile movement, particularly in understudied organisms like amphibians. We assessed the roles of morphological (snout‐vent length and relative leg length) and performance (maximal jump distance) traits in shaping the free movement paths, measured through fluorescent powder tracking, in three anuran species, Pacific treefrog (*Hyliola regilla*), Western toad (*Anaxyrus boreas*), and Cascades frog (*Rana cascadae*). We standardized the measurement of these traits to compare the relative role of species' innate differences versus physical traits in shaping movement. Innate differences, captured by species identity, were the most significant factor influencing movement paths via total movement distance and path sinuosity. Relative leg length was an important contributor but significantly interacted with species identity. Maximal jump performance, which was significantly predicted by morphological traits, was not an important factor in movement behavior relative to species identity. The importance of species identity and associated behavioral differences in realized movement provide evidence for inherent species differences being central to the dispersal and movement of these species. This behavior may stem from niche partitioning of these sympatric species, yet it also calls into question assumptions generalizing anuran movement behavior. These species‐level effects are important in framing differences as past research is applied in management planning.

## 1 INTRODUCTION

Movement and dispersal of organisms across a landscape are key drivers of ecosystem function. This repositioning of individuals is central to processes such as nutrient cycling, population genetics, and cross‐boundary subsidies (Baguette, Blanchet, Legrand, Stevens, & Turlure, 2013; Bohonak & Jenkins, 2003; Carlson, Mckie, Sandin, & Johnson, 2016; Massol et al., 2011). Animal dispersal, broadly defined by Bowler and Benton (2005) as “movement between habitat patches,” is often characterized by resource or environmentally directed movements that are strongly influenced by organismal condition and involves shifts in habitat during natural growth and development (e.g., green sea turtles: Arthur, Boyle, & Limpus, 2008; migrant passerines: Chernetsov, 2006; anadromous salmonids: Kahler, Roni, & Quinn, 2001). Therefore, the study of dispersal requires a life history framework to better understand the integrative effects of movement to a species’ ecology across ontogenetic transitions (Benard & McCauley, 2008; Whitlatch et al., 1998).

Juvenile dispersal is a movement phase that is often a major life history transition, requiring individuals to navigate new and unfamiliar habitats (Clobert, Massot, & Le Galliard, 2012; Popescu, Brodie, Hunter, & Zydlewski, 2012). For example, amphibian and macroinvertebrate metamorphosis, with subsequent transition from aquatic to terrestrial habitats, represents a distinct shift in habitat in which movement ability is dependent on newly acquired morphological traits, such as legs and wings (Bilton, Freeland, & Okamura, 2001; Rowe & Ludwig, 1991; Smith & Green, 2005). While these morphological traits allow for simple quantification, the link between these emerging traits, behavior, and dispersal ability is not clearly established (Sekar, 2012). We posit that the ability to disperse into new and unfamiliar habitats may be more strongly regulated by behavior than morphology (Dyck & Baguette, 2005). Individual variation in the ability to move and the choice of how to move could have critical implications for survival and fitness (Bonte et al., 2012).

Behaviors, such as movement timing, directionality, and microhabitat preference, strongly regulate the potential movement ability of an organism (Rehage & Sih, 2004). For example, a highly mobile hummingbird (green hermit, *Phaethornis guy*) will increase movement distance and path sinuosity to remain in their preferred forested habitats while homing through a complex composition landscape (Hadley & Betts, 2009). Organisms experiencing unfamiliar habitats, such as newly metamorphosed amphibians experiencing terrestrial habitats for the first time, must rely heavily on innate behaviors to guide their movements (Popescu et al., 2012; Rothermel, 2004). Thus, the integration of morphological and behavioral trait response may be the guiding principle in shaping juvenile orientation and dispersal (Patrick, Harper, Hunter, & Calhoun, 2008).

Movement is not only important for the fulfillment of resource needs and life history transitions, but also can be an important mechanism to allow for species coexistence (Jeltsch et al., 2013). Differences in movement behavior of ecologically similar spadefoot toad species provide a mechanism to reduce competition (Székely, Cogălniceanu, Székely, & Denoël, 2017). High‐elevation pond‐breeding amphibian communities rely on shared habitats during a narrow breeding window (Lannoo, 2005). These results in overlapping development and emergence creating a scenario with intense intra‐ and interspecific competition: Variability in movement behaviors between species and within cohorts could reduce this competition pressure (Harper & Semlitsch, 2007).

In this study, we use a behavioral and morphological framework to understand amphibian movement ecology. Further, we apply this conceptual model to juvenile life stages, a critically understudied life history stage that coincides with transitional movement from aquatic to terrestrial habitats (Cline & Hunter, 2016; Ramírez, Bell, Germano, Bishop, & Nelson, 2017; Roe & Grayson, 2008). Laboratory‐based quantification of individual performance measures, such as jumping ability, speed, and endurance, has been commonly used as proxies for individual dispersal and natural movement (Binning, Shaw, & Roche, 2017; Llewelyn, Phillips, Alford, Schwarzkopf, & Shine, 2010; Louppe, Courant, & Herrel, 2016; Phillips, Brown, Webb, & Shine, 2006). Body size and morphology have also been important determinants of individual performance and in some cases dispersal (John‐Alder & Morin, 1990; Yagi & Green, 2017). Important measures have included leg length and body size to predict movement ability of juvenile amphibians (Gomes, Rezende, Grizante, & Navas, 2009; Tejedo, Semlitsch, & Hotz, 2000). We used an experimental approach to assess the roles of individual performance ability, morphological traits, and species‐specific intrinsic behavior in shaping realized movement. Understanding how juvenile amphibians, with limited experience, adjust to both new morphological traits and habitats will provide insights into potential constraints on dispersal and movement in a taxon of conservation concern (Pittman, Osbourn, & Semlitsch, 2014).

Our objective was to compare the role of morphological and species‐specific traits in shaping movement paths of amphibians in a transitional phase using three sympatric amphibian species, Pacific Treefrog (*Hyliola regilla*), Western Toad (*Anaxyrus boreas*), and Cascades Frog (*Rana cascadae*). To do this, we quantified athletic performance and morphometrics by measuring maximal jump distance, snout‐vent length, and leg morphology. We then used fluorescent powder tracking in a bare agricultural field to observe and quantify the free movement behavior of these individuals. These three species comprise the anuran community of high‐elevation ponds in the Cascade Mountains of Oregon with similar reproductive and emergence phenologies. By representing three distinct anuran families, each with their unique adaptations, we can compare the relative contributions individual performance and morphology with species‐level differences on movement. We hypothesized that during this transitional phase, variation in movements would be best explained by innate differences between species, whereas morphological and performance‐based variation would play a minor role.

## 2 METHODS

### 2.1 Collection and rearing

Egg masses of each species were collected from five breeding sites in the central Oregon Cascades between 1,800 and 2,050 m elevation during the summer breeding season of 2014. Individuals were collected from at least six separate clutches per species per population to reduce clutch effects. Embryos were pooled by species and reared to hatching in a temperature‐controlled environmental chamber set at 15°C with a 12L:12D photoperiod at Oregon State University. Within 8 hr of hatching, individuals were grouped by species and raised in an outdoor mesocosm array.

The outdoor mesocosm array consisted of 30, 120‐L HDPE plastic tubs filled with well water and stocked at a constant density of 30 individuals of a species per mesocosm. Outdoor mesocosms were located in the Willamette Valley at Oregon State University's Lewis‐Brown Horticulture Farm. Given these species exist across a gradient of hydroperiod conditions, we randomly assigned individuals to mesocosms with either a permanent or ephemeral hydroperiod to simulate more natural conditions and determine whether the larval environment had any latent effect on juveniles. Water volume in the permanent hydroperiod mesocosms (*n* = 15 tubs) was maintained at 100 L throughout the course of development, resulting in a density of 0.3 individuals/L. Water volume in the ephemeral hydroperiod mesocosms (*n* = 15 tubs) was reduced at a rate of 8 L every 5 days, beginning with 100 L water volume (0.3 individuals/L) and ending with 12 L water volume (2.5 individuals/L) over the course of 60 days. While part of the initial experimental design, larval mesocosm condition did not impact any quantified movement parameters and was instead included as a blocking variable in our analyses. Mesocosms were checked daily for juveniles emerging onto floating platforms starting at day 20 or at Gosner (1960) stage 42.

Upon emergence, animals were moved to a temperature‐controlled environmental chamber set at 20°C with a 12L:12D photoperiod at Oregon State University and maintained in 5‐L HDPE plastic containers grouped by mesocosm and fed wingless fruit flies ad libitum*.* Individuals were held for at least 10 days to ensure they had survived metamorphosis and were accepting food as juveniles.

### 2.2 Experimental design

We assessed maximal jump performance and movement behavior for each individual (total *n* = 175) on the same day. The experiment was blocked across seven trial days (24, 25, 27 September–1 October 2014). Twenty‐four individuals were assessed on a single trial day except for Day 7 when 33 animals were sampled. Each trial day included an equally representative sample of all three species (*n* = 8 per species). Logistical demands required all remaining individuals to be run on day 7 (*H. regilla*: *n* = 14, *A. boreas*: *n* = 12, and *R. cascadae*: *n* = 7).

Maximal jump performance was measured as the longest observed jump across two trials of four jumps with a minimum of two hours of rest in between trials (John‐Alder & Morin, 1990). Jump trials took place during the day between the hours of 10:00 and 17:00 on a cleaned, sterilized, and dry laboratory bench. Individuals jumped along the bench, stimulated with an approaching gloved hand, and gentle prods of the individual's posterior were used when animals stopped jumping for more than 2 s (Mitchell & Bergmann, 2016). After four jumps were recorded, animals were held in individual perforated plastic 1‐cup containers with moisten paper towels until their next measurement. After the conclusion of the jump trials, individuals were measured for snout‐vent length and mass. After at least 2 hr of additional rest time, individuals were transported to Hyslop Field Lab where we measured free movement behavior in a plowed and smoothed dirt field. This environment allowed for a standardized surface for all individuals and acted to limit the effect of microhabitat on movement. Movement measurements began after sunset at 20:00 (around nautical twilight) on nights free of precipitation to standardize abiotic conditions as much as possible. We used only dim red lights during the releases to minimize the effect of artificial lighting on behavior. We implemented a staggered release schedule over 60 min and provided each individual 60 min of free movement. Each individual was placed at least 10 m away from the nearest individual to limit interactions from influencing movements. Individuals were lightly dusted with fluorescent tracking powder (ECO Pigments, Day‐Glo Color Corp.) and placed on a petri dish lid under a cover object for a 5‐min acclimation period. After acclimation, cover objects were gently removed, and individuals were given 60 min to freely move about the field. After 60 min, individuals were located using UV lights and their final position was marked. The first individual was recaptured at 21:05, we recaptured in the same sequence with the last recaptured at 22:05. The movement path of each individual during the trial was then observable via tracking the powder residue on the ground under UV illumination. We measured total path length and net distance from start using measuring tapes.

Measures of abiotic conditions for each night were measured using the AgriMet Weather Station (CRVO) onsite for nightly temperature, relative humidity, wind speed and direction, and 24‐hr precipitation history (Table 1). Upon completion of both jump and movement trails, all individuals were humanely euthanized using MS‐222 and preserved in 70% ethanol. Preserved animals were then photographed on a gridded and scaled background for measurement of average rear leg lengths in ImageJ (Schneider, Rasband, & Eliceiri, 2012).

**Table 1:** The ambient conditions measured at AgriMet Weather Station (CRVO) during field measurements across trial days

Variable	Average	*SD*
Temperature (°C)	15.5	1.6
Wind speed (km/hr)	5.3	2.7
Humidity (%)	80.5	12.1
Daily rain (mm)	0.25	0.25

## 3 STATISTICS

### 3.1 Morphology and jumping ability

To investigate the factors that influenced maximal jump performance, we fit a linear regression model to the data from all species and individuals (Table 2). Since there are inherent differences in jumping ability between species, the response variable of maximal jump performance was centered by subtracting the mean and scaled by dividing by the standard deviation for each species to account for this variation while making the general athletic ability of each individual comparable between species (Emerson, 1978). The predictor variables of this model were species, snout‐vent length (SVL), and relative leg length (RLL). Larval mesocosm conditions and trial day were included as blocking factors. Interactions of species with all morphometric measurements were included to allow for species‐specific effects on predictor variables. Both the values for SVL and RLL were centered and scaled (subtracting the mean and dividing by the standard deviation) within each species to again account for species‐specific morphological differences. Relative leg length was calculated as the ratio of average rear leg length (mm) to snout‐vent length (mm). All effects discussed are back‐transformed onto the original response variable scale.

**Table 2:** The explicit statistical models used in the analysis of each section

Section	Response	Model
Morphology and Jumping Ability	Maximal Jump	Spp* + RLL + SVL + Spp*:RLL + Spp*:SVL + trial_day^■^ + mesocosm^■^
Morphology and Movement	Total Distance, Path Shape	Spp* + RLL + SVL + Spp*:RLL + Spp*:SVL + trial_day^■^ + mesocosm^■^
Jumping Ability and Movement	Total Distance, Path Shape	Spp* + Max_Jump + Spp*:Max_Jump + trial_day^■^ + mesocosm^■^

*Note.* Factor variables are denoted by an asterisk. Factor variables used as blocks in the analysis are denoted by a solid square. Interaction terms are listed as the two variables separated by a colon.

### 3.2 Morphology and movement

Our analysis of movement path shape included response variables of total movement distance and straightness index (Benhamou, 2004). We performed an analysis of movement paths using a multivariate linear regression model with both response variables fit simultaneously (Table 2). The variable of total movement distance was log‐transformed to correct for non‐normality. The path sinuosity measure was determined by the ratio of total distance moved to net distance from start to finish. This index measure of path straightness ranges from 0 to 1; movement paths closer to 1 approached straight lines and paths closer to zero exhibited increasing sinuosity. This model similarly used species, SVL, RLL, larval mesocosm condition, and trial day as predictor variables, with SVL and RLL centered and scaled (subtracting the mean and dividing by the standard deviation) for each species. We included interaction terms for species and all morphometric variables to allow for species‐specific effects of predictor variables. Using this model, Pillai's trace tested for significant effects of predictor variables on movement paths. In addition, we compared the relative proportion of total variance explained by predictor variables using a measure of *ƞ* ^2^_partial_ (partial eta‐squared).

### 3.3 Jumping ability and movement

Performance measures such as jumping ability have been useful proxies for individual fitness and dispersal ability (Mitchell & Bergmann, 2016; Pough, 1989). To avoid multicollinearity, we did not include jump performance with the morphometric predictor variables in the analysis of movement path. Yet jump performance could have an important connection to the free movement of these amphibians. To explore the relevance of maximal jump performance, we built an additional multivariate linear regression model with the same movement path response variables and replaced morphometric measurements with jump performance (Table 2). Maximal jump distances were centered and scaled (subtracting the mean and dividing by the standard deviation) for each species to correct for inherent differences in ability. The predictor variables of this model included species and the interaction of species with jump performance. In addition to jump performance and species, we included larval mesocosm condition and experimental night as blocking variables. We again tested variables for significance using Pillai's trace and compared the relative proportion of total variance explained by predictor variables using a measure of *ƞ* ^2^_partial_.

All statistical tests were performed in R (version 3.4.0; R Core Team, 2017) using packages “car” (Fox & Weisberg, 2002), “heplots” (Fox, Friendly, & Monette, 2016), and “ggplot2” (Wickham, 2009) for analysis and creation of graphs.

## 4 RESULTS

**Figure 1:**
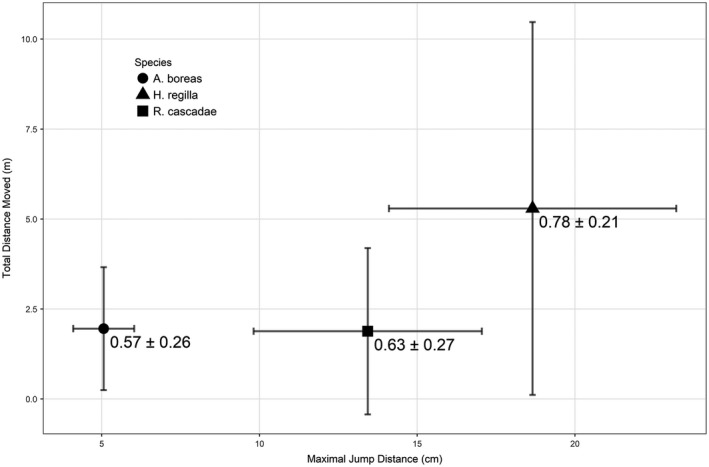
The average maximal jump distance and total movement distance for *Anaxyrus boreas*, *Hyliola regilla*, and *Rana cascadae*. Error bars represent one standard deviation. Numerical values on the plot represent the average path straightness index with one standard deviation

The three species included in our study had distinct jumping abilities and movement behaviors (Figure 1). The average maximal jump distance for *A. boreas* was 5.06 cm (*n* = 60, *SD* = 0.97), which was shorter relative to *R. cascadae* and *H. regilla,* with average maximal jumps of 13.4 cm (*n* = 53, *SD* = 1.88) and 18.7 cm (*n* = 60, *SD* = 4.55), respectively. The movement paths for *A. boreas* were short and sinuous (total distance: $\overset{¯}{x}$ = 1.95 m, *SD* = 1.7; straightness index: $\overset{¯}{x}$ = 0.57, *SD* = 0.26). *Rana cascadae* movement paths closely resembled those of *A. boreas* (total distance: $\overset{¯}{x}$ = 1.88 m, *SD* = 2.3; straightness index $\overset{¯}{x}$ = 0.63, *SD* = 0.27). The movements of *H. regilla* were the longest and straightest of the three species tested (total distance: $\overset{¯}{x}$ = 5.29 m, *SD* = 5.1; straightness index: $\overset{¯}{x}$ = 0.78, *SD* = 0.21).

### 4.1 Morphology and jumping ability

**Figure 2:**
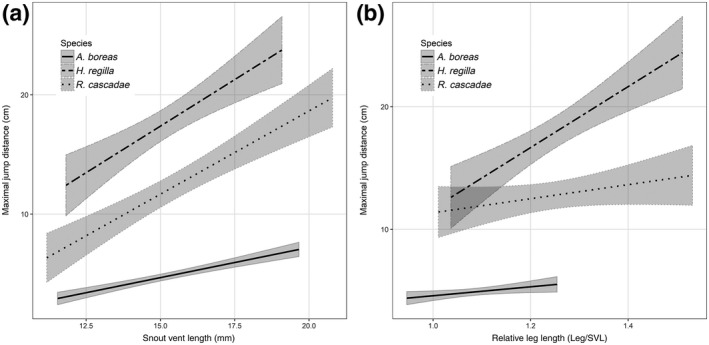
Model predicted effects of snout‐vent length (SVL) and relative leg length (RLL) on the maximal jump distance of three anuran species: *Anaxyrus boreas*, *Hyliola regilla*, and *Rana cascadae*. The left panel shows the species‐specific impact of SVL on maximal jump performance. The right panel shows the species‐specific impact of RLL on maximal jump performance. Values are back‐transformed onto their original scale. Shaded regions indicate 95% confidence intervals

From a biomechanical perspective, there is evidence of a strong relationship between morphometric measures and jump performance (Emerson, 1978). Our results found that across all species, the snout‐vent length was the most significant predictor of maximal jump performance (*F*_1,158_ = 72.2, *p* < 0.0001; Table 3). This effect of SVL on jumping performance indicated an increase in maximal jumping distance with increasing SVL (Figure 2). This was in addition to a significant interaction of species and SVL (*F*_2,158_ = 3.36, *p* = 0.037), where the strength of this effect of SVL and jumping performance was significantly smaller for *A. boreas*. An individual's relative leg length also had a significant direct effect on maximal jump performance (*F*_1,158_ = 4.85, *p* = 0.029) and significant interaction with species (*F*_2,158_ = 3.12, *p* = 0.047). Individuals with relatively longer legs for their body length showed increased maximal jump performance (Figure 2). The relationship of RLL and maximal jump performance was significantly stronger in *H. regilla*.

**Table 3:** ANOVA table with type III sum of squares for the analysis of maximal jump performance with morphological characteristics and species identity predictors

Response	Source	*df*	*SS*	*F*	*p*
Maximal jump performance	(Intercept)	1	0.602	1.314	0.253
	Species	2	0.003	0.003	0.997
	Relative leg length	1	2.222	4.847	0.029*
	Snout‐vent length	1	32.859	71.675	<0.001*
	Mesocosm condition	1	0.086	0.187	0.666
	Trial day	6	3.435	1.249	0.284
	Spp:RLL	2	2.884	3.146	0.046*
	Spp:SVL	2	3.008	3.281	0.040*
	Residuals	157	71.975		

*Note.* Mesocosm condition and trial day are included as blocking variables. *p*‐Values were considered significant at levels less than 0.05 and are marked with an asterisk.

### 4.2 Morphology and movement

**Figure 3:**
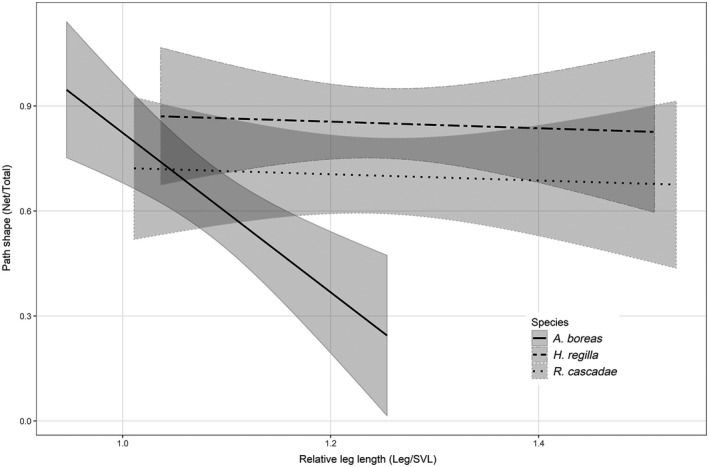
Model predicted effects of relative leg length (RLL) on path shape in *Anaxyrus boreas*, *Hyliola regilla*, and *Rana cascadae*. The index of path shape indicates straightness of movement with lower values representing more tortuous paths. Values are back‐transformed onto their original scale. Shaded regions indicate 95% confidence intervals

**Figure 4:**
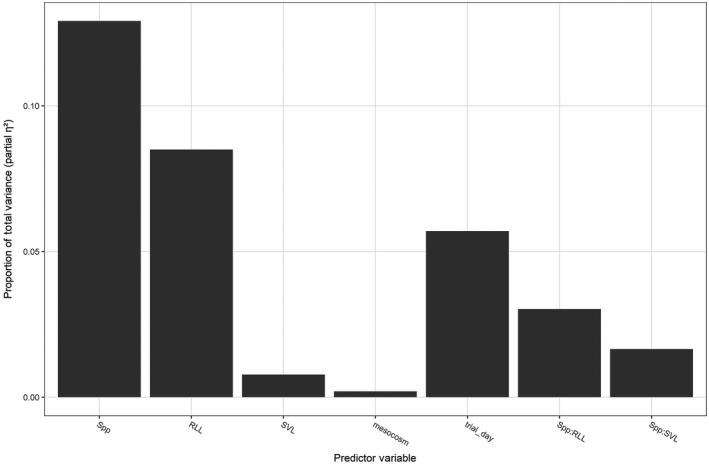
The relative proportion of total variance (*ƞ* ^2^_partial_) explained by the modeled predictor variables on movement path variables of total movement distance and path shape using predictors of species identity and morphometric traits

Movement paths were significantly affected by species identity (Pillai's trace = 0.258, *F*_4,314_ = 4.85, *p* < 0.0001) and RLL (Pillai's trace = 0.085, *F*_2,156_ = 4.85, *p* = 0.001), as well as the interaction between these two variables (Pillai's trace = 0.030, *F*_4,314_ = 4.85, *p* = 0.046; Table 4). The specific effects on path sinuosity involved the interaction of species identity and RLL. Path shape became more sinuous for *A. boreas* as RLL increased, yet RLL had minimal impact on path shape for the other two species (Figure 3). While both species identity and the morphometric trait of RLL were significant predictors in our model, our analysis revealed that species identity explained a higher proportion of the relative proportion of total variance in movement paths (species identity = 12.9%, RLL = 8.5%; Figure 4). Only species identity was a significant predictor of total movement distance. Total movement distance was only impacted by species identity with *H. regilla* increasing total distance by 210% (95% CI: 135%–325%) compared to the other two species.

**Table 4:** MANOVA table with Pillai's trace test statistic for the multivariate analysis of path with responses of total distance and path sinuosity

Response	Source	*df*	*V*	Approx. *F*	Num *df*	Den *df*	*p*
Total distance path sinuosity	(Intercept)	1	0.232	23.560	2	156	<0.001*
	Species	2	0.258	11.634	4	314	<0.001*
	Relative leg length	1	0.085	7.244	2	156	0.001*
	Snout‐vent length	1	0.008	0.609	2	156	0.545
	Mesocosm condition	1	0.002	0.158	2	156	0.854
	Trial day	6	0.114	1.581	12	314	0.096
	Spp:RLL	2	0.060	2.448	4	314	0.046*
	Spp:SVL	2	0.033	1.319	4	314	0.263

*Note.* Predictor variables included morphological and species identity. Mesocosm condition and trial day are included as blocking variables. *p*‐Values were considered significant at levels less than 0.05 and are marked with an asterisk.

### 4.3 Jumping ability and movement

**Figure 5:**
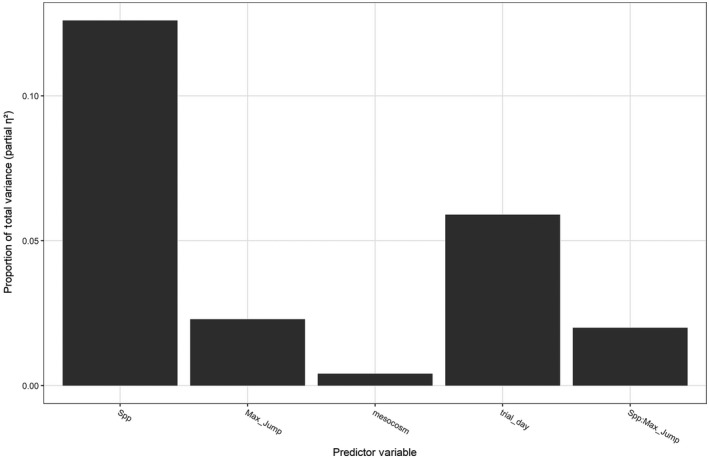
The relative proportion of total variance (*ƞ* ^2^_partial_) explained by the modeled predictor variables on movement path variables of total movement distance and path shape using predictors of species identity and standardized maximal jump performance

Our analysis of movement path variables with both species identity and maximal jump performance indicated that species identity was also the only significant predictor of movement path variables (Pillai's trace = 0.252, *F*_4,320_ = 11.53, *p* > 0.0001; Table 5). When effects of species identity on the univariate responses were examined, the differences were exhibited in the paths of *H. regilla* with 210% longer total movement distances than the other species (95% CI: 135%–325%) and straighter paths than the other species with an increase of 0.22 in the straightness index (95% CI: 0.13–0.31). This importance of species identity is similarly apparent by explaining 12.6% of the relative proportion of total explained variance (*ƞ* ^2^_partial_, Figure 5).

**Table 5:** MANOVA table with Pillai's trace test statistic for the multivariate analysis of path with responses of total distance and path sinuosity

Response	Source	*df*	*V*	Approx.* F*	Num *df*	Den* df*	*p*
Total distance Path sinuosity	Species	2	0.252	11.532	4	320	<0.001*
	Maximal jump	1	0.023	1.865	2	159	0.158
	Mesocosm condition	1	0.004	0.327	2	159	0.721
	Trial day	6	0.118	1.672	12	320	0.072
	Spp:Jump	2	0.040	1.629	4	320	0.167

*Note.* Predictor variables included maximal jump performance and species identity. Mesocosm condition and trial day are included as blocking variables. *p*‐Values were considered significant at levels less than 0.05 and are marked with an asterisk.

## 5 DISCUSSION

Our study found an important interaction between morphology and behavior on the movement and dispersal potential within three amphibian species. As predicted, morphology was a strong predictor of juvenile frog jump performance. Larger‐bodied individuals were able to jump farther regardless of species. Species‐level differences, however, emerged during our field trials as a key determinant of free movement paths. Powder tracking allowed for parameterization of path straightness and total distance. Straightness was predicted by both morphology (RLL) and species identity. Total distance traveled, however, was not a function of morphology, but solely of species identity. These results indicate that a reliance on morphology alone to understand movement is overly simplistic. Broad generalizations of movement based on organismal measures exclude important species‐specific behaviors (Hillman, Drewes, Hedrick, & Hancock, 2014). Integration of morphology and species‐specific differences is particularly important as independent factors influencing sinuosity or total distance moved together result in realized movements. Only through this integration is a holistic understanding of movement and dispersal potential possible.

To bridge existing research on performance measures with movement behavior, we first quantified a common amphibian performance metric: maximal jump performance. Our results found evidence that both individual body size and relative leg length are important predictors of maximal jump performance in these three species, which supports past work in anurans showing a strong morphological basis of jumping performance (Boes & Benard, 2013; Johansson, Lederer, & Lind, 2010; Tejedo et al., 2000). Individuals with longer bodies (SVL) increased their maximal jumping distance. Jump performance also improved with increasing leg length relative to body size (RLL). These two factors were significant variables in individual maximal jump performance, though their relative importance did vary between species.

When individuals were moved out of the laboratory environment to the field, RLL continued to demonstrate importance in our measurement of free movement paths. However, species identity was an overwhelmingly important factor in determining movement. We found distinct species differences in movement behaviors such as path sinuosity and total distance traveled. Though it is important to note that the observed effects did not quantify some additional sources of variation including population or clutch specific effects. Our hydroperiod treatment also did not control for density or water level independently as these are inherently linked in natural ephemeral ponds. Even with these added sources or variation, we observed distinct movement differences that we interpret as being ecologically relevant, with our most morphologically and behaviorally mobile species (*H. regilla*) occupying a generalist niche throughout their range relative to the other two species tested (*A. boreas* and *R. cascadae*) (Lannoo, 2005). Aspects of movement biology and niche breadth have actually been proposed as explaining differences in species ranges (Penner & Rödel, 2017).

Our results fit into a niche partitioning perspective nicely. These three amphibian species are sympatric in the Cascade Mountain Range during their aquatic life history stages. Differences in natal dispersal capacity and behavior could provide an important mechanism for reducing overlap during the transition of these species to terrestrial habitats. The importance of species identity in our analysis does encompass a variety of potential aspects of biology that contribute to these movement behaviors. For example, *A. boreas* may have less motivation to move in an effort to find moist microhabitats, as toads are more tolerant of dry conditions (Gatten, 1987). Species‐specific morphology and locomotion type can also impact movement ability (Petrović, Vukov, & Tomašević Kolarov, 2016). Variation in juvenile and adult terrestrial habitat requirements may also impact these movement behaviors (Lannoo, 2005). Ranid species such as *R. cascadae* often rely on habitats in, or surrounding, lentic areas and may not commonly move large distances after metamorphosis (Semlitsch & Bodie, 2003). Other studies have found that land cover can also impact movement behavior (Cline & Hunter, 2014; Stevens, Polus, Wesselingh, Schtickzelle, & Baguette, 2004; Youngquist & Boone, 2014) which could be differentially responded to by species. The synchrony and timing of metamorphosis, which varies between species, could influence movement behavior for species that rely on conspecific density to trigger mass emergence from pond margins. As individual size was also an important factor, species or individuals that emerge earlier could grow faster thereby providing additional potential for movement. Regardless, there is very little known about the juvenile dispersal and movement of these species outside of occasional observational notes.

The variation in habitat, life history, and ecology captured by species identity can be the result of many biological mechanisms. As such, the ability to generalize movement and dispersal across anuran species is potentially called into question. The diversity of species movement makes knowledge gaps extremely concerning; in a biological database of European amphibians, 26 species of anurans (52%) and 23 species or urodeles (63.8%) lacked movement data (Trochet et al., 2014). Conservation planning for any data deficient species would require managers to assume similar responses to amphibians more generally (Woltz, Gibbs, & Ducey, 2008). Our results indicate that species identity plays an overarching role in shaping the movement behavior of juvenile amphibians. More research should be directed at identifying the important mechanisms that drive movement behavior and management decisions should avoid the assumption that all anuran species exhibit the same morphological and/or behavioral capacity to move and disperse.

The limited predictive ability of maximal jump performance on movement potential also indicates that we need to move away from laboratory‐based performance measures into realistic movement scenarios or in situ animal tracking (e.g., Cline & Hunter, 2014, 2016; Ramírez et al., 2017; Roe & Grayson, 2008; Zamora‐Camacho, 2018). Performance measures have an important role as proxies for fitness in controlled studies on morphology and physiology (Pough, 1989). Our results confirm the strength of this relationship as we found a significant predictive power of morphology on maximal jump performance. Research has additionally extended these relationships by incorporating ecological diversification to account for species differences in performance (Gomes et al., 2009). The opportunity to generalize individual physiology and laboratory performance into movement seems to provide an option to address deficient field data (Hillman et al., 2014), yet the measures do not always relate to movement paths (Walton, 1988). Performance ability is only expressed in movement paths and subsequent dispersal when combined with individual movement behavior (Yagi & Green, 2017). Further, some free movement endpoints, such as total distance traveled, were not strongly tied to morphology or jump performance. We found that after 60 min of movement in the field, the point where a frog ended up was largely determined by species identity, or more succinctly, species‐specific behavioral choices. Such information further supports our key finding that performance measures and morphology need to be used in combination with realized movement behavior to establish their relative importance for movement more generally in amphibians.

Juvenile amphibians are also an important and critically understudied life stage to focus additional research efforts. For a taxonomic group that is of serious conservation concern, it is essential that we direct research toward a more holistic understanding of their ecology and behavior. For instance, we could potentially learn a great deal about how amphibians respond to novel environmental and/or climatic conditions from how they respond to novel habitats through ontogeny. Immediately after metamorphosis, juvenile amphibians have very limited experience with which to influence their movement behavior in the terrestrial environment. To survive in this novel environment, juveniles must appropriately respond to and learn from a suite of selective pressures through choices in behaviors like movement, refuge use, and foraging. Even beyond individual responses, differential mortality of dispersing juveniles could have strong selective pressures on the connectivity of populations (Delgado, Ratikainen, & Kokko, 2011). Information on the innate behavior and learning processes that impact movement, dispersal, and subsequent survival of juveniles will be essential information for conservation analyses and planning.

## 6 CONCLUSION

Our results suggest that individual morphology and associated performance measures can impact aspects of organism movement, but species‐specific behavioral traits were the driving factor of free movement paths in these juvenile amphibians. Performance measures can be useful proxies for some aspects of an organism's biology, yet we must be critical of their predictive use as they may not always correspond to natural movements. To properly develop our understanding of the ecology of amphibian movement in their natural habitats, we must coalesce the associated physiological and performance information and expand it to include real‐world movement and behavior. Our understanding of movement and its drivers, particularly during major life history transitions, offers an advancement in our understanding of species’ interactions with their environment and identifying aspects of habitat that are important across life stages.

## CONFLICT OF INTEREST

Authors have no conflict of interest.

## AUTHORS’ CONTRIBUTIONS

EB, TG, and AM conceived the research question. EB and LT collected and reared animals. EB collected data and performed the analysis with contributions from LT. EB wrote the manuscript with significant assistance from LT, AM, and TG. All authors contributed critically to the drafts and gave final approval for publication.

## Data Availability

Associated data will be published and publicly accessible on ScholarsArchive@OSU https://doi.org/10.7267/C247DZ48X.
